# Supplementary figures and images for: A Strainer-Based Platform for the Collection and Immunolabeling of Porcine Epidemic Diarrhea Virus-Infected Porcine Intestinal Organoid
Source: Int J Mol Sci. 2023 Oct 27;24(21):15671. doi: 10.3390/ijms242115671 (PMC10650080; doi:10.3390/ijms242115671)

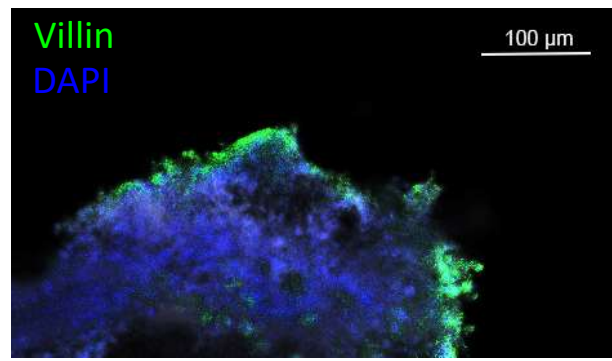

**Figure S1.** Villin labeled porcine intestinal organoids.

Supplement: Supplementary file 1 [file ijms-24-15671-s001.zip › ijms-2661179-supplementary.pdf]
